# Molecular Pathophysiology of Renal Tubular Acidosis

**DOI:** 10.2174/138920209787581262

**Published:** 2009-03

**Authors:** P.C.B Pereira, D.M Miranda, E.A Oliveira, A.C. Simões e Silva

**Affiliations:** Pediatric Nephrology Unit, Department of Pediatrics, School of Medicine – Federal University of Minas Gerais (UFMG), Belo Horizonte, MG, Brazil

**Keywords:** Renal tubular acidosis, acid-base homeostasis, molecular physiology, tubular transport, gene mutations.

## Abstract

Renal tubular acidosis (RTA) is characterized by metabolic acidosis due to renal impaired acid excretion. Hyperchloremic acidosis with normal anion gap and normal or minimally affected glomerular filtration rate defines this disorder. RTA can also present with hypokalemia, medullary nephrocalcinosis and nephrolitiasis, as well as growth retardation and rickets in children, or short stature and osteomalacia in adults. In the past decade, remarkable progress has been made in our understanding of the molecular pathogenesis of RTA and the fundamental molecular physiology of renal tubular transport processes. This review summarizes hereditary diseases caused by mutations in genes encoding transporter or channel proteins operating along the renal tubule. Review of the molecular basis of hereditary tubulopathies reveals various loss-of-function or gain-of-function mutations in genes encoding cotransporter, exchanger, or channel proteins, which are located in the luminal, basolateral, or endosomal membranes of the tubular cell or in paracellular tight junctions. These gene mutations result in a variety of functional defects in transporter/channel proteins, including decreased activity, impaired gating, defective trafficking, impaired endocytosis and degradation, or defective assembly of channel subunits. Further molecular studies of inherited tubular transport disorders may shed more light on the molecular pathophysiology of these diseases and may significantly improve our understanding of the mechanisms underlying renal salt homeostasis, urinary mineral excretion, and blood pressure regulation in health and disease. The identification of the molecular defects in inherited tubulopathies may provide a basis for future design of targeted therapeutic interventions and, possibly, strategies for gene therapy of these complex disorders.

## INTRODUCTION

The term Renal Tubular Acidosis (RTA) defines many disorders characterized by metabolic acidosis, secondary to defects in renal tubular reabsorption of bicarbonate (HCO_3_^−^) and/or in urinary excretion of hydrogen (H^+^), while glomerular function is little or not affected [1-6]. All forms of RTA present hyperchloremic metabolic acidosis, with normal anion gap and are chronic diseases with significant impact on the quality of life of affected patients when left untreated, possibly leading to growth failure, osteoporosis, rickets, nephrolithiasis and even renal insufficiency [1-6].

Defects in proximal bicarbonate reclamation or distal acid secretion give rise to the respective clinical syndromes of proximal or distal RTA [1-6]. These disorders can be primary, originating from genetic defects on tubular transport mechanisms [7], or secondary to systemic diseases and to adverse drug reactions [8-12]. The familial conditions exhibit distinct inheritance patterns. Distal RTA can be transmitted as either an autosomal dominant or an autosomal recessive trait, whereas isolated proximal RTA usually occurs as an autosomal recessive disease [6,7,13]. In the past few years, the molecular genetic strategies of positional cloning and candidate gene analysis have been combined to identify the genes responsible for these inherited conditions [6,13]. This review will summarize the mechanisms of acid-base regulation by the kidney and the current understanding of the genetic causes of primary inherited RTA. It will, in addition, evaluate the ability of known functional and biochemical properties of these mutant proteins to explain the pathophysiology of associated renal acidification defects.

## BRIEF OVERVIEW OF RENAL ACID-BASE HOMEOSTASIS

The kidney plays two major roles in acid-base homeostasis. First, the filtered bicarbonate load (approximately 4000 mmol/day) must be reabsorbed, mainly in the proximal tubule and beyond in the loop of Henle and distal nephron. This reclamation process in the proximal tubule minimally requires the following: hydrogen (H^+^) secretion of an equivalent amount *via *the luminal Na^+^/H^+^ exchanger (NHE-3) and the vacuolar H^+^-ATPase; luminal carbonic anhydrase type IV (CAIV) and cytosolic carbonic anhydrase type II (CAII); and basolateral bicarbonate exit through the electrogenic Na^+^-dependent bicarbonate cotransporter (NBC-1) [2,14-17]. Second, the kidney must regenerate new bicarbonate (approximately 50 ± 100 mmol/ day) in the process of acid-secretion, mainly in the collecting ducts, to match the amount of newly produced acid load by systemic metabolism [18,19]. In addition to sufficient buffer in the lumen, this process requires activities of several transport proteins of the acid secreting α-intercalated cells, including the luminal vacuolar H^+^-ATPase, CA II, and the basolateral chloride-bicarbonate exchanger, AE1 [18,20,21].

### Proximal Tubular Bicarbonate Reabsorption

HCO_3_^-^ is freely filtered at the glomerulus and approximately 80 to 90% of this is reabsorbed in the proximal tubule [6]. In the tubular lumen, HCO_3_^-^ combines with H^+^ in a reaction catalyzed by CA IV, which is bound to the luminal membrane of proximal tubular cells [2,14,15]. This reaction produces carbonic acid, which is promptly converted to CO_2_ and H_2_O. The resulting CO_2_ rapidly diffuses into the tubular cells and is combined with water to produce intracellular H^+^ and HCO_3_^-^. This intracellular reaction is catalyzed by CA II. HCO_3_^-^ is then cotransported with Na^+^ into blood (with a probable stoichiometry of 3 HCO_3_^–^ to 1 Na^+^) [6] *via *the NBC-1, located on the basolateral cell membrane. The intracellular H^+^ produced by CA II is secreted into the tubular lumen predominantly *via *the NHE-3, situated on the luminal membrane [6,15,22]. This transport process is called facilitated diffusion and depends on the sodium concentration gradient generated by the action of a basolateral membrane Na^+^-K^+^-ATPase. It should be mentioned that there is minimal net acid excretion in the proximal tubule, since most of the H^+^ secretion is coupled with HCO_3_^-^ reabsorption [6,13]. The small amount of remaining H^+^ will be buffered by phosphate as titratable acid. HCO_3_^-^ reabsorption is influenced by luminal HCO_3_^-^ concentration and pH, luminal flow rate, peritubular pCO_2_, and angiotensin II [2,6,17].

Proximal tubular cells are capable of generating “extra” bicarbonate through the deamination of glutamine to glutamate, then forming α-ketoglutarate and eventually glucose. This metabolic process produces HCO_3_^-^ and NH_4_^+^: the former reclaimed *via *the basolateral membrane and the latter secreted into the tubular lumen. This pathway can be upregulated in states of chronic acidosis [3,6,15].

The main mechanisms of proximal tubular bicarbonate reabsorption are displayed in Fig. (**1**).

### Distal Tubular Hydrogen Secretion

One of the important roles of the collecting duct segment of the nephron is acid secretion, combined with reclamation of the approximately 10% of filtered HCO_3_^-^ that is not reabsorbed by more proximal nephron segments [18]. The average omnivorous human diet in the `Western' world is rich in protein, and generates 1±1.5 mmol hydrogen/kg body weight each day [23]. Urinary acid excretion is therefore essential, and urine pH can drop as low as 4.5. The α-intercalated cell is the main responsible for hydrogen secretion into the urine. In humans at least, hydrogen pumps, called H^+^-ATPases, mainly carry out hydrogen secretion [18,19,23]. H^+^-ATPases are present at high density on the luminal membrane of α-intercalated cells [18]. Studies in nonhuman mammals show that these H^+^-ATPases are also present within specialized intracellular tubulovesicles close to the membrane, allowing additional pumps to be recruited to the membrane quickly in to response to stimuli, such as systemic acidosis, for example [23]. These cells secrete H^+^ into the lumen of the distal tubule and collecting duct not only *via *H+-ATPase but possibly also by an exchanger, H^+^/K^+^-ATPase [7,10]. In addition, the normal function of the luminal H^+^-ATPase in α-intercalated cells is coupled, in a poorly understood manner, to the electroneutral transport of HCO_3_^-^ back across the basolateral surface into the interstitial fluid, and hence to blood. The transporter responsible for this activity in renal α-intercalated cells is the Cl^-^/HCO_3_^-^ exchanger AE1 [7,20,21]. The AE1 exchanger is homologous with the red cell anion exchanger known as ‘band 3’ (eAE1) [6,24]. After the red cell, the kidney is the next richest source of this protein (kAE1) [24]. Proton secretion varies with systemic pH and it is also aldosterone-dependent and voltage-dependent [24].

Once secreted, net urinary elimination of H^+^ depends on its buffering and excretion as titratable acid (mainly phosphate - HPO_4_^2−^ + H^+^ ↔ H_2_PO_4_^−^), and excretion as NH_4_^+^ [24]. Notably, the production of NH_4_^+^ from glutamine by the proximal tubule, and its subsequent excretion in the urine, also generates ‘new’ bicarbonate, which is added to plasma [24]. Availability of phosphate as a buffer depends on its filtration, whereas NH_4_^+^ depends on normal function of the proximal tubule, as well as a complex process of secretion, reabsorption, and secretion again along the nephron [24]. The final secretory step for NH_4_^+^ excretion is ‘diffusion trapping’ in the collecting duct. Anything that interferes with H^+^ secretion in the collecting duct will reduce diffusion trapping and cause a decrease in excretion of both H^+^ and NH_4_^+^ [6,24]. As previously mentioned, chronic metabolic acidosis stimulates renal NH_4_^+^ synthesis and excretion [3,6,15].

Fig. (**2**) shows renal acidification process in α-intercalated cells of the distal nephron.

## CLASSIFICATION AND CLINICAL FEATURES OF RENAL TUBULAR ACIDOSIS

Clinically, RTA is characterized by a normal anion gap, hyperchloremic metabolic acidosis, and associated failure to thrive secondary to growth failure as well as anorexia [13]. Polyuria and constipation can also be seen, although neither may be apparent in the neonatal period [13]. Hyperchloremic metabolic acidosis in pediatric practice is most often associated with diarrheal disease. Both diarrhea and RTA result in hypokalemia. For this reason, in a young infant with diarrhea and underlying RTA, the true diagnosis may be obscured. Thus, inordinately slow resolution of hyperchloremic metabolic acidosis following diarrheal disease should suggest the possibility of an underlying primary RTA [13].

Beyond the difficulties inherent in delineating RTA, RTA can be subcategorized into different disorders with distinctly diverse prognoses [13]. The diagnostic cataloguing of RTA is based on the underlying pathophysiology. The current model of how the nephron reabsorbs HCO_3_^−^ and secretes H^+^ has led to a clinical and functional classification of proximal (tubule) versus distal (tubule and collecting duct) forms of RTA [24]. Thus, the main types of RTA are proximal (or type 2) RTA and distal (or type 1) RTA. Type 3 RTA is a mixed type RTA that exhibits both impaired proximal HCO_3_^–^ reabsorption and impaired distal acidification, and more disturbingly osteopetrosis, cerebral calcification and mental retardation [4]. Hyperkalemic (or type 4) RTA is a heterogeneous group of disorders that is characterized by low urine NH_4_^+^, which is probably caused by the hyperkalemia or by aldosterone deficiency or defective signaling [4].

In distal RTA, distal nephron net acid secretion is impaired. This leads to a high urine pH, even in the presence of systemic acidosis [2,4]. However, there is often no metabolic acidosis and the blood bicarbonate concentration is normal, so-called ‘incomplete’ distal RTA, and a defect in renal acid excretion must be demonstrated by a failure to lower urine pH below 5.5 following an NH_4_Cl load or a modified furosemide test [2,6,24]. Acquired distal RTA is often secondary to autoimmune diseases, such as Sjogren’s syndrome [6,24]. Inherited distal RTA can be essentially of three types: autosomal dominant distal RTA (the commonest form) and autosomal recessive distal RTA with and without sensorineural deafness [24]. In the complete forms of both dominant and recessive distal RTA bone disease is common (rickets or osteomalacia), as well as nephrocalcinosis (often) complicated by renal stone disease. The occurrence of renal stones is attributed to the combination of hypercalciuria, low urinary citrate excretion (due to systemic and intracellular acidosis) and high urine pH, all favouring calcium phosphate stone formation. Hypokalaemia, another characteristic feature, is less troublesome than in the acquired autoimmune form of distal RTA, but it can become symptomatic, especially if a thiazide diuretic is prescribed to reduce hypercalciuria [24]. In recessive distal RTA, some patients suffer from sensorineural deafness, which can be late in onset [24].

Conceptually, the proximal tubule is charged with the task of reclaiming filtered HCO_3_^-^ (~ 85% of the total) [13]. Failure of this process leads to reduction in systemic base, resulting in metabolic acidosis – proximal RTA [13]. Proximal RTA typically manifests as part of a generalized defect of proximal tubule function, namely the renal Fanconi’s syndrome (with glycosuria, low molecular weight proteinuria, urinary phosphate wasting, hypophosphataemia and hypouricaemia) [24]. Isolated proximal RTA occurs rarely and usually presents as growth retardation in childhood. Like distal RTA, it can be divided into three types: autosomal recessive proximal RTA with ocular abnormalities, autosomal recessive proximal RTA with osteopetrosis and cerebral calcification, and autosomal dominant proximal RTA [24]. Autosomal recessive proximal RTA with ocular abnormalities is the commonest form of isolated and inherited proximal RTA, but even this is rare. Ocular abnormalities include band keratopathy, glaucoma and cataracts [24]. Short stature is usual; dental enamel defects, mental retardation, hypothyroidism, abnormal pancreatic function and basal ganglia calcification are also features [24,25]. In inherited CA II deficiency, isolated proximal RTA presents with osteopetrosis (due to impaired osteoclast function), cerebral calcification and variable mental retardation [26]. Although this form of inherited RTA is clinically more proximal in type, it can also present with a mixed proximal and distal phenotype, which reflects the presence of CA II in cells all along the renal tubule.

Type 3 RTA can be caused by recessive mutation in the *CA2* gene on chromosome 8q22, which encodes CAII [4] or could involve direct interaction between CA II and the NBC1 [27] or Cl^–^/ HCO_3_^–^ exchanger, *SLC26A6* [4,28].

The causes of type 4 RTA include various types of adrenal failure or pseudohypoaldosteronism type 1 (PHA1) due to defects in the mineralocorticoid receptor or the epithelial Na^+^ channel, all characterized by salt loss and hypotension [4]. A similar picture may be seen in obstructive uropathy or drug induced interstitial nephritis [4]. Furthermore, a number of drugs may impair signalling in the renin–angiotensin-aldosterone system and cause hyperkalemia and metabolic acidosis (e.g. potassium sparing diuretics, trimethoprim, cyclo-oxygenase inhibitors, angiotensin converting enzyme inhibitors) [4]. Lately, much interest has been given to a group of rare autosomal dominant diseases characterized by hyperkalaemia and acidosis and age-related hypertension [4]. In spite of hypervolaemia, aldosterone is not low and the disorders have been collectively termed pseudohypoaldosteronism type 2 (PHA2) [4].

## INHERITED FORMS OF DISTAL RENAL TUBULAR ACIDOSIS

Inherited forms of distal RTA have three variants: autosomal dominant and autosomal recessive with or without deafness. Dominant disease typically presents more mildly in adolescence or adulthood, and recessive variant occurs in infancy/early childhood, where growth retardation is common [6]. In Table **1** we can see the chromosome mapping of distal RTA.

### Autosomal Dominant Distal RTA (Distal RTA Type 1a )

Distal RTA occurs with the greatest frequency as an isolated defect, often transmitted as an autosomal dominant trait [13]. In few reported families, the presence of the disorder in several generations suggests an autosomal dominant transmission. Although clinical findings are not different from those observed in autosomal recessive or sporadic cases, in these patients the disease may be diagnosed later (in adolescence or adulthood) [6] or manifest with milder symptomatology.

Autosomal dominant distal RTA has been found to be associated in several kindred with mutations in the *SLC4A1 *gene encoding the CI^-^/HCO_3_^-^ exchanger, AE1 [15].

#### The Electroneutral Anion Exchanger (AE1)

The Cl^-^/HCO_3_^-^ anion exchanger, AE1, is a glycoprotein encoded by a gene (*SLC4A1*) present on chromosome 17 q21-22. *SCL4A1* gene is a member of the *SLC4 *family comprising 10 genes of which 8 encode bicarbonate ion transporters [6,24,29]. AE1 is an integral membrane glycoprotein containing a long cytoplasmic N-terminus (~ 400 amino acids), 12–13 transmembrane domains (responsible for anion transport and dimerization), and a short cytoplasmic C-terminus (~ 35 amino acids) [30,31]. It is predominantly expressed in the erythrocytes (eAE1) and in the kidney (kAE1).

kAE1 is a truncated isoform of eAE1 with lacking of 65 amino acids at the N-terminus owing to the use of differential transcription and translation start sites [32]. This extra NH2-terminal sequence confers additional roles for eAE1, including facilitation of red cell metabolism and maintenance of erythrocyte structural stability *via *interaction with a glycolytic enzyme complex and cytoskeletal elements, respectively [6]. kAE1 mediates an electroneutral exchange of chloride for bicarbonate at the basolateral membrane of acid secreting α-intercalated cells of the distal nephron and collecting duct [32,33]. This ion exchanger promotes the reabsorption of bicarbonate into the blood. Therefore, eAE1 defect results in morphological changes of red blood cells (RBC) while kAE1 abnormality leads to distal RTA [32].

The physiological role of kAE1 in the regulation of distal nephron acid secretion is well established. In the acidification process of the distal nephron, basolateral kAE1 mediates Na^+^-independent, electroneutral Cl^-^/HCO_3_^-^ exchange, allowing HCO_3_^-^ to exit the α-intercalated cells in concert with apical H^+^ secretion *via *the vacuolar H^+^-ATPase.

#### AE1 Gene (SLC4A1) Mutations

Because of the expression of AE1 in two different cells (RBC and α-intercalated distal tubular cells) with distinct functions, AE1 mutations show pleiotrophic effects resulting in two distinct and seemingly unrelated phenotypes: hereditary spherocytosis (or other forms of erythrocyte abnormalities) and distal RTA [31]. The largest group of mutations in human AE1 is associated with autosomal-dominant red cell dysmorphologies (hereditary spherocytosis – HS; and Southeast Asian ovalocytosis - SAO), where renal acid-base handling is normal [6]. AE1 mutations also result in distal RTA, because the defect in AE1 affects anion Cl^-^/HCO_3_^-^ exchanger at the basolateral membrane of the α-intercalated cells in the distal nephron [31].

SAO, a well-known erythrocyte disorder, is caused by a deletion of 27 bp in codons 400-408 in exon 11 (Ex11D27) of AE1 leading to a lack of 9 amino acids in the protein, which is inactive for anion transport.

How can be explained either the absence of red cell abnormalities in patients with distal RTA or the rarity of defects in distal urinary acidification in patients with hematological disorders, when, in both circumstances, mutations in the same *SLC4A1 *gene are present? [15]. One exception is the homozygous AE1 mutant V488M (Band 3 Coimbra; GTG → ATG), which presents with severe anemia and renal acidification defect [34,35].

The majority of AE1 mutations apparently cause only erythroid abnormalities without renal phenotype. Most cause autosomal dominant forms of HS and are not encountered in homozygous form, suggesting embryonic lethality [7].

Dominant HS-associated AE1 mutations are generally not associated with distal RTA. Conversely, distal RTA-associated AE1 mutations are also not commonly accompanied with HS. Whereas HS missense mutations are distributed throughout AE1 cytoplasmic and transmembrane domains, distal RTA mutations are restricted to AE1’s transmembrane domain. Although, the almost complete segregation between mutations associated with HS and with distal RTA is not fully understood [7].

Autosomal dominant distal RTA was first associated with exon 14 nucleotide substitutions encoding missense mutations in residue 589 (R589), in which the wild-type Arg is converted to His, Ser, or Cys [30,36]. A single base change alters the identical AE1 residue, R589, in eight of the ten reported kindred with dominant distal RTA, supporting the importance of this residue in the normal acidification process. R589 lies at the intracellular border of the sixth transmembrane domain of the protein, adjacent to K590. These basic residues are conserved in all the known vertebrate anion exchanger isoforms and are thought to form part of the site of intracellular anion binding. Arginine at this position is conserved in all vertebrate AE proteins, indicating its functional importance [37].

Three different mutations at this position (R589C, R589H, and R589S) were found in autosomal dominant distal RTA and two *de novo* R589H mutations have also been reported [30,32,36]. A high prevalence of AE1 R589 mutations and the presence of at least two *de novo* mutations at this position suggest that codon 589 (CGC) is a “mutational hotspot” of AE1*. *The mechanism of recurrent mutations probably involves methylation and deamination altering cytosine (C) to thymine (T) in the CpG dinucleotides [37].

Another missense mutation alters serine to phenylalanine at position 613 [36] within the adjacent transmembrane loop, evidencing the importance of this region of the protein. A further complex mutation results in a C-terminally truncated AE1 protein lacking the last 11 amino acids [29].

#### AE1 in Autosomal Recessive Distal RTA

Recent gene studies have shown that some of the AE1 mutations are responsible for autosomal recessive distal RTA in several countries in Southeast Asia; these patients may be homozygous for the mutation or be compound heterozygotes of two different AE1 mutations, one of which is usually the SAO mutation [38,39]. The evaluation of the AE1 G701D mutation has provided the first explanation for how any distal RTA-associated AE1 mutation might cause the disease [40].

Recessive distal RTA appears to result from the absence or a very marked deficiency of chloride-bicarbonate exchange activity in the basolateral membrane of the distal α-intercalated cell. In the case of the G701D mutation this occurs because the mutant protein is totally dependent on the presence of glycophorin A (GPA) for its movement to the cell surface. GPA is a glycosylated protein that is associated with band 3 and has a single span across the erythrocyte membrane [38]. Expression in *Xenopus *oocytes demonstrated that GPA completely rescues the cell surface movement of the G701D mutant band 3 to normal levels. This contrasts with normal band 3, which moves to the cell surface even in the absence of GPA, although GPA further enhances this movement. Red blood cells contain GPA but GPA is absent from the kidney, hence individuals homozygous for the G701D mutation have normal levels of band 3 in their red cells. It is proposed that, in homozygotes, the mutant G701D protein does not reach the basolateral membrane of the α-intercalated cell, but is turned over within the cell. In SAO/G701D compound heterozygotes, the SAO protein is presumed to reach the cell surface, but since it is inactive in anion transport, it acts as if it were a band 3 null allele [38].

### Autosomal Recessive Distal RTA with Deafness (Distal RTA Type 1b)

Recessive forms of distal RTA are related to mutations in the proton pump in α-intercalated cells. The gene involved (*ATP6V1B1*) is located on chromosome 2q13, and encodes the B1-subunit of H^+^-ATPase expressed apically on α-intercalated cells and also in the cochlea and endolymphatic sac [4,23].

In the human cochlea, the H^+^-ATPase appears to be required to maintain normal endolymph pH [6] given that the very high potassium concentration (approximately 150 mmol/l) in this closed compartment is not normally accompanied by alkalinity of the endolymph [23]. *ATP6V1B1 *expression has also been observed in the male genital tract (with acidification requirement for sperm maturation) [29].

Clinical findings, other than deafness, are identical to those present in patients with sporadic or autosomal recessive distal RTA and normal hearing. There is great variation in the presentation of deafness, from birth to late childhood, it is progressive and does not respond alkali therapy [15]. The defects in B1 cause irreversible hair cell damage in human cochlea because of ambient electrolyte and pH abnormalities [29].

Screening for mutations in this gene revealed fifteen different mutations in kindred. The majority of these mutations are likely to disrupt the structure, or abrogate the production, of the normal B1 subunit protein [29].

#### The Human Vacuolar H^+^-ATPase

The vacuolar-type proton ATPase (V- or H+-ATPase) is a multisubunit pump that is essential for normal acidification of intracellular vesicular structures. In each individual cell, H^+^-ATPases may function in a variety of distinct but essential cellular processes. However, the mechanisms by which cells regulate the intracellular trafficking, final destination and activity of these proton pumps are unclear [41].

The H^+^-ATPases are composed of two structural domains (membrane-bound V_0_ and cytoplasmic or peripheral V_1_) each formed of multiple subunits (a–e and A–H, respectively), which are responsible for ATP hydrolysis and proton transport, respectively [6,23]. The mammalian H^+^-ATPase is presumed to be similar to that of yeast (in which most of the structural studies have been performed) [23].

### Autosomal Recessive Distal RTA with Preserved Hearing (Distal RTA Type 1c)

Individuals without hearing defects carry mutations at chromossome 7 q33-q34. The defective gene is *ATP6V0A4*, which encodes a kidney-specific a4 isoform subunit of H^+^-ATPase. The involvement of the a4 subunit in distal RTA shows that it must be essential for proper proton pump function in the kidney [29], but its role is not totally clear.

Site-directed mutagenesis studies of the yeast ‘a’ subunit ortholog Vph1p (the ‘a’ subunit in proton pumps localized to the yeast vacuole) have yielded some potential functions [42]. Some mutations showed that this subunit is important for the assembly of the proton pump, whereas other mutations had greater effects on ATPase activity and proton transport. These studies suggest that the ‘a’ subunit is important for both assembly and function of the pump [29,42].

## INHERITED FORMS OF PROXIMAL RENAL TUBULAR ACIDOSIS

Proximal RTA is caused by a reduction in bicarbonate reabsorption at the proximal tubules, resulting in low renal bicarbonate threshold. The most common proximal RTA in children is secondary to Fanconi Syndrome [2,43]. Rarely, RTA might also be consequence of an inherited or sporadic primary renal disorder.

The acquired proximal RTA follows exposure to drugs or some toxins and the etiopathogenesis is still unknown [2]. Among drugs that cause Fanconi Syndrome are gentamicin, cisplatin, ifosfamide, and sodium valproate [6]. In addition, some hematologic and autoimmune conditions, such as myeloma and Sjogren syndrome respectively, might also course with proximal RTA.

The proximal RTA resulting from Fanconi Syndrome is frequently part of a systemic syndrome. Among systemic disorders that result in RTA, the inheritance pattern is usually autosomal recessive. Some of these disorders are cystinosis, tyrosinaemia, galactosaemia, Fanconi-Bickel syndrome and others (Table **2**) [44]. These syndromes are a heterogeneous group of disorders, which genes are mapped in many chromosome regions.

The RTA non-related to Fanconi Syndrome is a rare disorder and might be sporadic, autosomal dominant or autosomal recessive. The autosomal recessive disorder is associated with ocular abnormalities, frequently coursing with mental retardation. Other clinical features are short stature, dental enamel defects, pancreatitis, and basal ganglia calcification [45]. Loss-of-function mutations in the gene that codifies the NBC-1, the* SLC4A4 *gene, were first identified in two Japanese subjects with proximal RTA associated with cataracts, glaucoma and band keratopathy [46]. NBC-1 is formed by 1,035 amino acids; it contains ten transmembrane domains and two cytoplasmic termini, and it is present in kidney, brain, eye, pancreas, heart, prostate, epididymis, stomach, and intestine. In the kidney, NBC-1 is expressed mainly at the basolateral membrane of the proximal tubule. At least two genes encode the NBC proteins. Mutations were identified in the human NBC-1 gene (*SLC4A4*) mapped at chromosome 4p21 [47,48].

Another interesting candidate gene for proximal RTA is the *TASK *gene*. *TASK2-potassium channel is a member of the tandem-pore domain potassium channel family and is located in pancreas, placenta, lung, small intestine, colon and kidney. TASK2 seems to be important to bicarbonate absorption in renal proximal tubules. Knockout mice for *TASK2* gene course with metabolic acidosis associated with low bicarbonate levels [49]. However, no mutation in these genes was yet identified in individuals with proximal RTA.

Other inherited form of proximal RTA is the one resulting from mutations in the gene *CA2* that encodes CAII. The carbonic anhydrases (CA) are member of a family of zinc metalloenzymes that catalyzes the hydration of CO_2_. The human CA2 maps to the chromosome region 8q22. In the kidney, the majority of CA activity is attributable to CA II, which is localized in proximal tubular cells and in α-intercalated cells of the cortical and outer medullary collecting tubules [50]. Due to their localization, this RTA course with some proximal and distal components. In terms of clinical aspects, this RTA present osteopetrosis, cerebral calcification and different levels of mental retardation.

The autosomal dominant proximal RTA was originally described in a large Costa Rican family [51,52], consisting of nine individuals presenting growth retardation and osteomalacia. No gene was found to be associated with this clinical presentation. Recently, another family with isolated proximal RTA inherited as an autosomal dominant disease was described [53]. The father and all four children had RTA with blood bicarbonate levels of 11-14 mEq/L and urine pH of 5.3-5.4 and all presented high bicarbonate fractional excretion. In terms of clinical aspects, they course only with short stature without other organ dysfunction. This family was investigated at the following genes: *CA II, CA IV, CA XIV, NCB1, Na^+^/H^+^ exchanger (NHE-3), NHE-8*, the regulatory proteins of *NHE3, NHRF1 and NHRF2* and the *Cl-HCO^-^_3_ exchanger, SLC26A6.* However, no mutation was found in any of the candidate genes studied. The study of these families might clarify other mechanisms involved in renal bicarbonate balance and a genome wide investigation of a pool of these families might result in interesting findings.

## INHERITED FORMS OF RENAL TUBULAR ACIDOSIS TYPE 3

Type 3 RTA is a mixed type that exhibits both impaired proximal HCO_3_^-^ reabsorption and distal acidification. The condition is due to an inherited deficiency of CAII caused by a recessive mutation in the *CA2* gene on chromosome 8q22, which encodes this widely expressed enzyme [4,6]. The expression of CAII is affected in bone, kidney (in both proximal and distal nephron segments, explaining the mixed acidosis) and brain.

The mechanisms that underlie the clinical picture in type 3 RTA, apart from much slower conversion of carbonic acid to and from bicarbonate, apparently also involve direct interaction between CA II and the kidney NBC1 [27] or Cl^–^/ HCO_3_^–^ exchanger, *SLC26A6 *(a plasma membrane Cl^–^/ HCO_3_^–^ exchanger with a suggested role in pancreatic HCO_3_^–^ secretion) [4,28]. Mutation of the identified CAII binding site reduced *SLC26A6* activity, demonstrating the importance of this interaction [28].

Patients with this deficiency exhibit osteopetrosis and cerebral calcification, as well as a mixed RTA with proximal and distal components [29]. This association of osteopetrosis and RTA is known as Guibaud-Vainsel syndrome or marble brain disease. Osteopetrosis is a condition of increased bone density, but also augmented bone fragility, leading to increased fracture risk, plus intracerebral calcification, intellectual impairment, growth failure, and facial dysmorphism. Excess bone growth leads to conductive deafness and can also cause blindness through compression of the optic nerve [6].

There is a considerable degree of heterogeneity, both in the predominance of proximal or distal acidosis and in the osteopetrotic phenotype [6]. In different kindred, mild or severe mental retardation has also been described.

Different mutations in *CA2* gene have been described; for example, the common ‘Arabic’ mutation, consisting of loss of the splice donor site at the 5’ end of intron 2 [6,29].

## INHERITED FORMS OF HYPERKALEMIC RENAL TUBULAR ACIDOSIS

Type 4 RTA is a heterogeneous group of disorders associated with hyperkalemia due to aldosterone deficiency or impairment in aldosterone molecular signaling.

Type 4 RTA might result from a PHA1. Some clinical aspects associated are hyponatremia, hyperkalemia, and elevated plasma aldosterone and plasma renin activity. The inheritance might be autosomal dominant or autosomal recessive [54]. The autosomal dominant is a frequent and mild kidney disorder without any other organ involvement [55]. This disorder seems to be associated to loss-of-function mutations in the mineralocorticoid receptor gene, the *MRL* gene. MRL-knockout mice develop symptoms of pseudohypoaldosteronism. In humans clinical presentation varies from non-symptomatic to important neonatal sodium loss. The recessive inheritance is associated to sodium transport defects in all aldosterone target tissues, not only kidney, but also colon, lungs, salivary and sweat glands. The recessive disorder is more severe and salt wasting is normally more pronounced. However, both types of inheritance might result in the same degree of natriuresis, hyperkalaemia and metabolic acidosis.

Other inherited cause of type 4 RTA includes hyperkalaemia associated with hypertension and low or normal levels of plasma aldosterone [56-58]. This syndrome is called pseudohypoaldosteronism type 2 (PHA2), or Gordon’s syndrome, which results in a renal aldosterone resistance inherited as an autosomal dominant pattern [6]. Mutations in the gene of two isoforms of WNK serine-threonine kinases, *WNK4* and *WNK1 *genes, were identified in patients with PHA2 [59]. WNKs are serine kinase proteins lacking a lysine residue at the active site, being the WNK type 1 a regulatory protein from *WNK 4.* *WNK4* is found in the distal nephron and controls the sodium and chloride reuptake and inhibits potassium efflux [6].

## CONCLUDING REMARKS

Renal tubular acidosis (RTA) is characterized by metabolic acidosis due to renal impaired acid excretion. In this review, we summarized our current understanding of the hereditary diseases caused by mutations in genes encoding transporter or channel proteins operating along the renal tubule. Further molecular studies of inherited tubular transport disorders may shed more light on the molecular pathophysiology of these diseases and may significantly improve our understanding of the mechanisms underlying renal salt homeostasis, urinary mineral excretion, and blood pressure regulation in health and disease. The identification of the molecular defects in inherited tubulopathies may provide a basis for future design of targeted therapeutic interventions and, possibly, strategies for gene therapy of these complex disorders.

## Figures and Tables

**Fig. (1):**
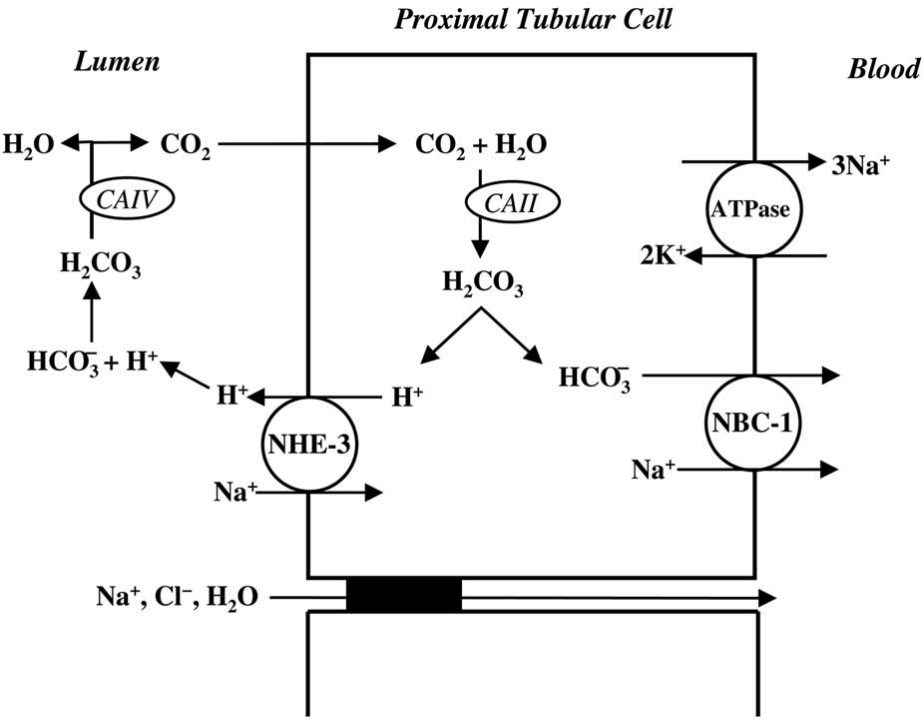
Schematic model of bicarbonate (HCO_3_^-^) proximal reabsorption. The intracellular carbonic acid (H_2_CO_3_^-^) dissociates into H^+^ and HCO_3_^-^ in a reaction catalysed by a cytoplasmic carbonic anhydrase (CAII). At the luminal membrane, H^+^ secretion is due to an especific Na^+^ – H^+^ exchanger (NHE-3), while, at the basolateral membrane, the 1 Na^+^ - 3 HCO_3_^-^ cotransporter (NBC-1) is responsible for HCO_3_^-^ transport to the peritubular capilar. The secreted H^+^ reacts with filtered HCO_3_^-^ to form luminal H_2_CO_3_, which is dissociated into H_2_O and CO_2_ by the action of membrane-bound carbonic anhydrase (CAIV). The generated CO_2_ diffuses back into the cell to complete the HCO_3_^-^ reabsorption cycle.

**Fig. (2):**
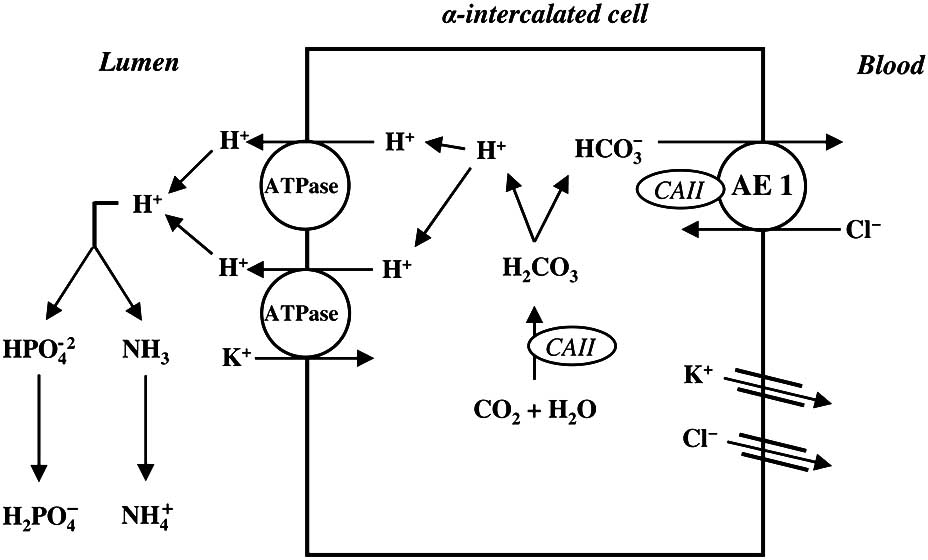
Schematic model of the α-intercalated cell and the H^+^ secretion in cortical collecting tubule. The α-intercalated cell is responsible for H^+^ secretion by a vacuolar H^+^-ATPase (main pump) and also by a H^+^-K^+^-ATPase. The luminal ammonia (NH_3_) buffers H^+^ to form nondiffusible ammonium (NH_4_^+^) and divalent basic phosphate (HPO_4_^-^) is converted to the monovalent acid form (H_2_PO_4_^-^) in H^+^ presence. Intracellularly formed HCO_3_^-^ leaves the cell *via* Cl^-^ - HCO^3^ ^-^ exchange, facilitated by an anion exchanger (AE1). Cytoplasmic carbonic anhydrase II (CA II) is necessary to secret H^+^.

**Table 1.** Chromosome Mapping of the Inherited Distal Renal Tubular Acidosis

Inherited Distal RTA	Gene	Mapping	Protein Encoded
Autosomal dominant	*SLC4A1*	Chromosome 17q21-q22	AE 1 exchanger
Autosomal recessive *(with deafness)*	*ATP6V1B1*	Chromosome 2q13	B1-subunit of H^+^-ATPase
Autosomal recessive *(with preserved hearing)*	*ATP6V0A4*	Chromosome 7 q33-q34	a4 isoform subunit of H^+^-ATPase

**Table 2.** Chromosome Mapping of the Inherited Fanconi Syndromes

Inherited Fanconi Syndromes	Gene	Mapping
Autosomal recessive	*SLC4A4*	Chromosome 4q21
Dent´s syndrome	*CLCN5*	Chromosome Xp11.22
Cystinosis	*SLC3A1* *SLC7A9*	Chromosome 2p21 Chromosome 19p13.1
Tyrosinaemia type 1	*FAH *gene	Chromosome 15q23-q25
Galactosemia	*GALT *gene	Chromosome 9p13
Wilson´s disease	*ATP7B *gene	Chromosome 13q14.3-q21.1

## ABBREVIATIONS

RTA: = Renal Tubulat Acidosis
NHE-3: = Na^+^/H^+^ exchanger
CAIV: = Carbonic anhydrase type IV
CAII: = Carbonic anhydrase type II
NBC-1: = Na^+^-dependent bicarbonate cotransporter
AE1: = Basolateral chloride-bicarbonate exchanger
eAE1: = Red cell anion exchanger
kAE1: = Kidney anion exchanger
CA2: = Carbonic anhydrase gene
PHA1: = Pseudohypoaldosteronism type 1
PHA2: = Pseudohypoaldosteronism type 2
RBC: = Red blood cells
HS: = Hereditary spherocytosis
SAO: = Southeast Asian ovalocytosis
GPA: = Glycophorin A
